# A spatio-temporal self-supervised meta-learning network with dynamic graph learning for traffic flow forecasting

**DOI:** 10.1371/journal.pone.0342520

**Published:** 2026-03-27

**Authors:** Qian Qiu, Yong Huang, Xiaoting Huang, Wei Zhou

**Affiliations:** 1 School of Traffic and Transportation, Guangxi Transport Vocational And Technical College, Nanning, China; 2 Guangxi Comprehensive Transportation Safety and Management Innovation Team, Nanning, China; 3 Guangxi Transportation Industry Safety and Emergency Response Technology Key Laboratory, Guangxi Transport Vocational And Technical College, Nanning, China; 4 Nanning Architectural Planning and Design Group Co., Ltd., Nanning, China; National University of Defense Technology, CHINA

## Abstract

Accurate traffic flow prediction is essential for alleviating urban congestion, improving road network efficiency, and sup-porting sustainable transportation systems. Existing spatio-temporal graph neural networks, however, often struggle to capture dynamically evolving spatial dependencies, effectively integrate spatio-temporal features, and generalize across diverse traffic scenarios. To address the challenges of accurately modeling complex spatio-temporal dependencies in traffic flow forecasting, this study proposes a Spatio-Temporal Self-Supervised Meta-Learning Network (SSML-Net), the model comprises spatial- and temporal-level learners, and integrates self-supervised meta-learning with meta-learning-driven feature fusion and gated coupling mechanisms to enhance spatio-temporal interaction and generalization capabilities. Comprehensive experiments on the PeMS datasets demonstrate that SSML-Net clearly outperforms traditional statistical approaches, deep temporal models, and spatio-temporal graph-based networks. The ablation study validated the effective-ness and necessity of the model’s core components, whilst the small-sample experiments demonstrated its robustness and generalisation capabilities under extreme data conditions. Concurrently, we expanded our training and evaluation experi-ments based on METR-LR and Beijing traffic data, further validating the model’s exceptional transfer learning capability and generalisation performance in cross-domain traffic forecasting scenarios. This approach not only achieved superior prediction accuracy but also substantially reduced model training costs. These results indicate that SSML-Net adapts to varying data scales and dynamic urban scenarios, providing a robust, adaptive, and high-precision spatio-temporal traffic flow prediction framework.

## 1. Introduction

With the acceleration of urbanization and the growing demand for resident mobility, the complexity of urban transportation systems continues to increase. How to achieve efficient and accurate traffic flow prediction has become a core issue in the development of Intelligent Transportation Systems (ITS) [1]. Traffic flow forecasting aims to make high-precision predictions of future traffic conditions—such as flow rates, speeds, or congestion levels—for specific time periods and road segments based on historical observation data, real-time traffic information, and external environmental factors [2]. Accurate prediction results not only provide decision-making support for traffic management authorities to implement proactive strategies such as signal control, route guidance, and congestion mitigation, but also serve as a vital reference for urban planning, logistics scheduling, and environmental governance [3].

Traffic flow forecasting is fundamentally a complex spatio-temporal sequence modeling problem characterized by dual spatial and temporal heterogeneity. In the spatial dimension, traffic patterns exhibit significant regional variations, and traffic conditions propagate through the road network with pronounced topological dependence. Adjacent road segments and distant nodes interact through the network structure [4]. In the temporal dimension, traffic flow exhibits both short-term localized fluctuations (such as instantaneous changes triggered by sudden incidents) and long-term cyclical patterns (such as the recurring occurrence of morning and evening rush hours) [5]. Meanwhile, compared to other time series forecasting tasks, traffic flow prediction presents unique challenges due to its complex spatiotemporal coupling relationships. These primarily encompass the distinctive topological connectivity patterns inherent to road networks and their continuously evolving spatiotemporal dynamic characteristics [6]. However, current mainstream deep learning models often treat spatial structure and temporal evolution as separate entities, making it difficult to generalize across different scenarios, time periods, and even cities. Therefore, effectively capturing the dynamic evolution patterns of traffic time series and the latent spatial dependencies within road network structures, while efficiently coupling spatio-temporal features to achieve high-precision predictions across scenarios and time periods, has become a key research focus and challenge in this field.

Early research primarily relied on statistical analysis methods for modeling and predicting traffic flow. These methods typically drew upon time series theory, assuming that traffic flow data exhibited certain stationarity or seasonality, and captured its variation patterns by fitting predefined mathematical models. Among these, the Autoregressive Integrated Moving Average (ARIMA) [7] model and its seasonal extension (SARIMA) [8] are widely applied, effectively handling linear dependencies and forecasting short-term traffic conditions. Additionally, Kalman filtering and its improved variants [9,10], along with historical averaging methods [11], have also been mainstream techniques, particularly suited for scenarios with sparse data or significant noise. Although statistical methods demonstrate certain effectiveness in modeling short-term time dependencies and offer strong interpretability, they also exhibit significant limitations. These methods heavily rely on linear assumptions and fixed parameters, making it difficult to capture the nonlinear and dynamic characteristics inherent in real traffic flows.

Subsequently, machine learning methods have been widely introduced into various complex system modeling and prediction domains, including lithium-ion battery state-of-health estimation [12], medical image analysis, and affective video content analysis [13], where they have demonstrated significant advantages in characterizing nonlinear dynamic evolution patterns; meanwhile, in the transportation domain, these techniques have been successfully applied to visual perception tasks [14], traffic sign detection [15], machine fault diagnosis [16], and intelligent 3D traffic accident reconstruction [17], in which machine learning models exhibit clear advantages in expressive power and flexibility compared with traditional statistical methods [18]. For instance, Chen et al. [19] proposed an activation-function–flexibly selected Kolmogorov–Arnold network to develop a universal magnetic core loss model, which comprehensively accounts for various materials and operating conditions while ensuring high predictive accuracy. Chen et al. [20] investigate the optimal placement of roadside Light Detection and Ranging for cooperative perception in intelligent transportation systems. A chance-constrained stochastic optimization model is developed to maximize expected detection accuracy under budget and traffic uncertainty constraints. Yan et al. [21] propose a novel arc detection method for the pantograph–catenary system of railway vehicles, namely a multimodal arc detection network based on denoising diffusion probabilistic models, which achieves excellent detection performance with only a small amount of data in complex railway environments.

In the field of traffic flow forecasting, traditional machine learning methods remain an important technical route in early research and engineering applications. For example, Support Vector Machines (SVM) [22,23] handle high-dimensional features through kernel function mapping, demonstrating strong generalization capabilities in short-term traffic forecasting. Random Forests [24] mitigate overfitting risks by integrating multiple decision trees, making them suitable for traffic flow data exhibiting pronounced periodicity and trendiness. Additionally, the K-means [25,26] clustering algorithm is employed to identify typical patterns in traffic conditions, thereby further enhancing the effectiveness of segmented forecasting. Despite this, traditional machine learning methods remain heavily reliant on manual feature engineering and exhibit limited modeling capabilities for spatio-temporal dynamic coupling relationships. They perform particularly poorly when handling large-scale road networks and long-term forecasting tasks.

Despite the substantial progress achieved in traffic flow forecasting, several critical research gaps remain. First, most existing spatio-temporal forecasting models rely on predefined or static graph structures, which fail to capture the dynamic and state-dependent evolution of spatial dependencies in real-world traffic networks. Second, current models often exhibit limited generalization capability when facing scenario shifts across different time periods, traffic states, or cities, largely due to their strong dependence on supervised learning and domain-specific data distributions. Third, although self-supervised and meta-learning techniques have been introduced in recent studies, they are usually explored independently, and a unified framework that jointly leverages self-supervised representation learning and meta-learning for spatio-temporal traffic prediction remains underexplored. Addressing these gaps is crucial for building robust, adaptive, and transferable traffic forecasting models in complex and non-stationary environments.

To address the challenges of accurately modeling complex spatio-temporal dependencies in traffic flow forecasting, this work proposes the Spatio-Temporal Self-Supervised Meta-Learning Network (SSML-Net). The core idea is to achieve precise and robust representation of dynamic traffic patterns through dual-layer decoupled modeling, meta-learning-driven parameter optimization, and adaptive spatio-temporal feature fusion. The overall architecture of SSML-Net features a parallel and decoupled design, consisting of a Spatial-level Learner and a Temporal-level Learner, which respectively capture the dynamic topological structure and multi-scale temporal variations of traffic data. Building upon this, a self-supervised meta-learning strategy is systematically integrated into both spatial and temporal dimensions, empowering the model with rapid adaptation and efficient generalization across diverse and changing traffic environments. In addition, a meta-learning-driven feature fusion and gated coupling mechanism is introduced, enabling deep interaction and dynamic integration of spatial and temporal representations. These innovations collectively equip SSML-Net with superior prediction accuracy and robustness in complex, non-stationary traffic scenarios. The main contributions of this research are as follows:

(1) We introduce a unified spatio-temporal prediction framework SSML-Net. It is a novel network architecture designed for traffic flow forecasting that jointly captures spatial topological dependencies, multi-scale temporal dynamics, and enables adaptive task-level generalization. By decoupling spatial and temporal modeling while simultaneously incorporating a meta-learning mechanism, SSML-Net achieves lightweight yet robust spatio-temporal joint modeling.
(2) We propose a state-aware spatial representation via dynamic graph learning. The spatial-level learner utilizes a gated graph update mechanism that adaptively adjusts graph connectivity based on real-time traffic states. This approach enables the model to capture dynamic, state-dependent spatial dependencies among road segments, providing robust spatial representation even under highly dynamic and non-stationary traffic environments.
(3) We design a Temporal-level Learner that fuses local convolutional features with global temporal context through cross-temporal attention. This enhances the model’s capability to represent both micro-level periodic variations and macro-level traffic evolution patterns, significantly improving forecasting accuracy across various time scales.
(4) We propose an integrated self-supervised meta-learning mechanism that jointly optimizes self-supervised representation learning and meta-learning processes. By leveraging abundant unlabeled traffic data to learn domain-invariant spatio-temporal features, this approach enables SSML-Net to achieve rapid adaptation and robust generalization in cross-domain scenarios, effectively transferring forecasting knowledge to unseen cities or regions even when only limited labeled data is available.

The remainder of this work is organized as follows: Section 2, “Related Work” formalizes the traffic forecasting task, providing problem definitions and a notation system; Section 3, “Methodology” systematically describes the overall architecture of SSML-Net, analyzing the design philosophy and implementation details of each key module; Section 4, “Experiments and Results Analysis” primarily introduces experimental design and results analysis. Section 5, “Conclusions”, summarizes the research work and discusses future research directions.

## 2. Related work

### 2.1. Deep learning-based prediction

In recent years, deep learning methods have been widely applied in traffic forecasting and have gradually become a mainstream research direction. Compared to traditional approaches, deep learning models can automatically extract high-level features from raw data without relying on complex manual feature engineering. They also possess robust nonlinear fitting capabilities and end-to-end learning capabilities [27]. In time series modeling, temporal convolutional networks (TCNs) [28] demonstrate high efficiency in modeling local temporal patterns. Recurrent neural networks (RNNs) [29] and their variants, such as long short-term memory (LSTM) [30] and gated recurrent units (GRU) [31], are widely employed to model the time-dependent nature of traffic flow. In particular, the introduction of Transformers [32] and their self-attention mechanism has significantly improved medium- and long-term prediction performance in traffic forecasting. In spatial modeling, convolutional neural networks (CNNs) [33] are employed to process the spatial grid structure in traffic data, effectively capturing local spatial correlations. However, real-world road networks exhibit complex topological structures and non-Euclidean characteristics, making it challenging for such methods to represent the connectivity and semantic dependencies between nodes adequately. This challenge prompted researchers to introduce Graph Convolutional Networks (GCNs) [34] and apply them to traffic forecasting, modeling road network structures explicitly. GCN defines node connections through adjacency matrices and integrates multi-order neighborhood information using message passing and aggregation mechanisms, significantly enhancing its ability to capture spatial dependencies [35]. However, while the aforementioned methods have made significant progress in modeling either the temporal or spatial dimension, they often struggle to simultaneously account for the dynamic spatial dependencies and complex temporal coupling characteristics within traffic flow data, failing to establish a unified spatio-temporal dynamic representation.

### 2.2. Spatiotemporal traffic flow prediction

To address the challenge of dynamically changing and complexly coupled spatial dependencies within road networks, researchers have proposed Spatio-Temporal Graph Neural Networks (STGNNs). By integrating graph convolutions with time series modeling, these networks not only explicitly capture the topological dependencies of road networks but also effectively capture the dynamic evolution patterns of traffic flows. Consequently, they demonstrate superior predictive performance in complex traffic scenarios. These models typically employ graph convolutional networks (GCNs) or graph attention networks (GATs) [36] to capture the spatial topology of road networks. They also integrate components such as recurrent neural networks (RNNs), temporal convolutional networks (TCNs), or Transformers to learn the temporal dynamics of traffic flow, thereby significantly enhancing prediction accuracy [37–38]. Li Y et al. [39] proposed the Diffusion Convolutional Recurrent Neural Network (DCRNN), which combines the diffusion process on directed graphs with an encoder-decoder architecture to simulate the propagation of traffic flow within road networks. This approach effectively captures the complex spatio-temporal dependencies inherent in traffic flow. Lu Bin et al. [40] constructed a dynamic weighted graph and combined it with a multi-head self-attention temporal convolutional network and an adaptive graph gating mechanism to effectively capture dynamic spatio-temporal dependencies in traffic flows. This approach achieved multi-step forecasting performance superior to baseline models on real urban traffic data. Zhao L et al. [41] proposed a temporal graph convolutional network (T-GCN) model that combines graph convolutional networks (GCNs) with gated recurrent units (GRUs), enabling effective capture of spatiotemporal dependencies in traffic data. The STGCN model developed by the Peking University team [42] innovatively combines graph convolutional networks (GCN) with gated temporal convolutions, providing an effective solution for capturing complex spatiotemporal dependencies in traffic networks. Ma et al. [43] proposed the Spatio-Temporal Transformer (STTLM) to achieve traffic flow prediction by decoupling and integrating complex spatio-temporal features through a pure Transformer architecture combined with a multi-level embedding mechanism. The Informer model proposed by Zhou et al. [44] significantly reduces the quadratic computational complexity and memory consumption of Transformers through three innovations: the ProbSparse self-attention mechanism, self-attention distillation, and a generative decoder. It achieves performance surpassing existing methods in long-sequence temporal prediction tasks. Huo et al. [45] proposed a novel hierarchical traffic flow prediction network. By synergistically integrating the Long-Term Transformer (LTT) with Spatio-Temporal Graph Convolution (STGC) and employing an innovative attention fusion mechanism, it effectively addresses the limitations of Graph Convolutional Networks (GCNs) in modeling long-term and short-term temporal relationships and excessive smoothing.

## 3. Methodology

In this section, we present the proposed Spatio-Temporal Self-Supervised Meta-Learning Network (SSML-Net) for traffic flow forecasting. The framework is designed to simultaneously capture spatial topological dependencies, multi-scale temporal dynamics, and adaptive task-level generalization, thereby achieving lightweight yet robust spatio-temporal joint modeling.

### 3.1. Overall framework of SSML-Net

Traffic flow forecasting requires simultaneously capturing complex spatial dependencies among road segments and diverse temporal dynamics across multiple time scales. To address this challenge, we propose a novel SSML-Net. The key idea is to decouple spatial and temporal modeling while introducing a self-supervised meta-learning mechanism that enables rapid adaptation and robust generalization under diverse traffic scenarios.

**Fig 1:**
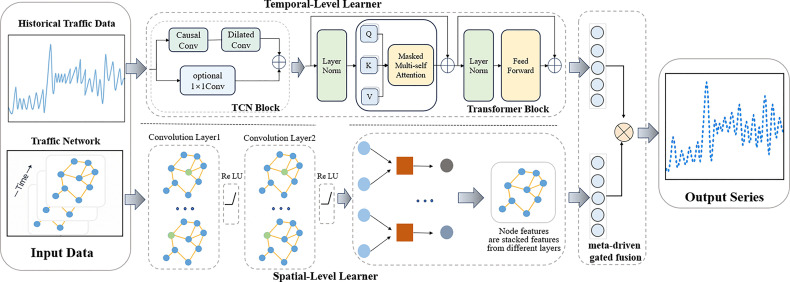
Overall framework of the SSML-Net prediction network.

As illustrated in Fig 1, the proposed SSML-Net is architected around two parallel and complementary learning modules: a Spatial-level Learner and a Temporal-level Learner. The former leverages a Graph Convolutional Network (GCN) to model dynamically evolving topological relationships across the road network. In parallel, the latter captures both short-term fluctuations and long-range dependencies in traffic sequences by integrating a Temporal Convolutional Network (TCN) with a lightweight Transformer encoder. These two streams of representations are adaptively fused via a meta-learning-driven gated fusion mechanism, which effectively couples spatial and temporal features in a context-aware manner. In contrast to conventional methods that often depend on static graph structures or single-scale temporal modeling, SSML-Net explicitly incorporates dynamic adjacency refinement, multi-scale temporal representation learning, and meta-learning-based parameter adaptation. This integrated design substantially enhances both prediction accuracy and model generalization under diverse traffic scenarios.

### 3.2. State-aware dynamic graph learning

**Fig 2:**
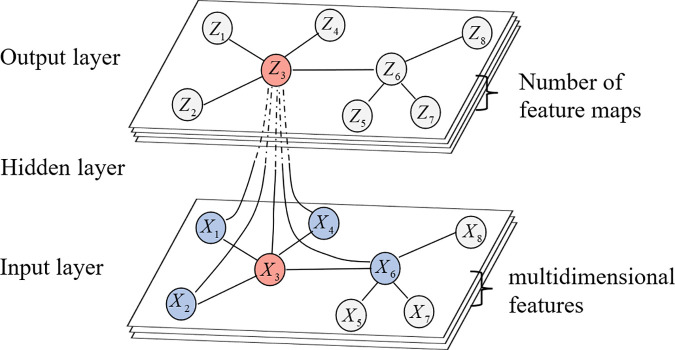
Illustration of the graph convolution process.


Spatial-level learner is designed to capture the evolving topological dependencies among road segments in a transportation network. Specifically, the learner first formalizes the transportation network as a directed graph G=(V,E,A), where V denotes the set of nodes, E represents the road connection relationships, and $A\in {\mathbb{R}}^{N\times N}$ corresponds to the adjacency matrix. Based on this representation, the Spatial Hierarchical Learner employs a Graph Convolutional Network (GCN) to model the adjacency relationships between nodes. By stacking L layers of convolutional operations, it progressively aggregates information from multiple levels of neighbors to effectively capture the spatial topological correlations between road segments (as shown in Fig 2). The general propagation rule of the GCN is [46]:

$$H^{\left(l+1\right)}=\sigma ({\tilde{D}}^{-\frac{\text{1}}{\text{2}}}\;\tilde{A}\;{\tilde{D}}^{-\frac{\text{1}}{\text{2}}}\;H^{\left(l\right)}\;W^{\left(l\right)})$$ (1)

where $\tilde{A}=A+I_{N}$ denotes the normalized adjacency matrix, $\tilde{D}$ denotes the corresponding degree matrix, $H^{\left(l\right)}$ represents the input feature matrix of the lth layer, $W^{\left(l\right)}$ denotes the trainable parameter matrix, σ(·) denotes a nonlinear activation function.

**Fig 3:**
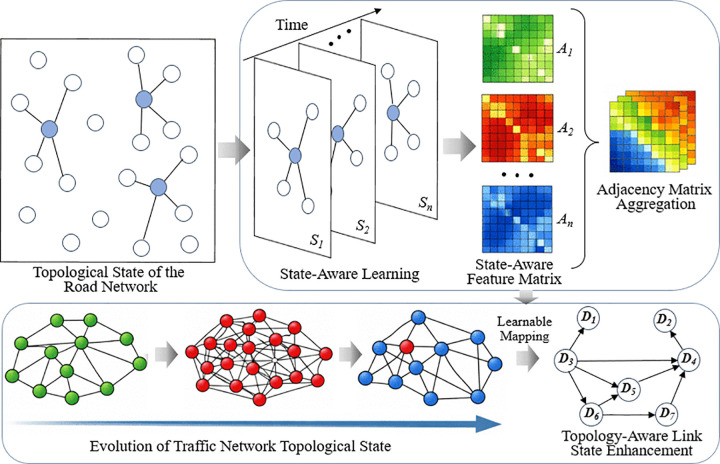
State-aware dynamic graph learning mechanism.


However, conventional static adjacency matrices fail to reflect temporal variations in connectivity caused by congestion propagation, accidents, or signal changes. To overcome this limitation, we propose a state-aware dynamic graph learning mechanism (as shown in Fig 3) that adaptively updates the adjacency structure according to real-time traffic states. The dynamic adjacency at time t is defined as:

$$\overset{dyn}{\underset{t}{A}}=f(Z_{t}W_{z}+S_{t})$$ (2)

where $Z_{t}$ denotes node embeddings, $W_{z}$ is a trainable weight matrix, and $S_{t}$ is a state-dependent adjustment term obtained from the current traffic observation $X_{t}$ and the previous hidden state $h_{t-1}$:


$$S_{t}=\emptyset (W_{s}\left(X_{t}\;,{\;h}_{t-1}\right)+{\;b}_{s})$$ (3)

where ∅(·) is an activation function.

Once $\overset{dyn}{\underset{t}{A}}$ is obtained, it is incorporated into a gated graph update mechanism to capture the dynamic topological evolution:


$$r_{t}=\sigma (W_{r}[X_{t}\;,{\;h}_{t-1}]+b_{r})$$ (4)

$$\;u_{t}=\sigma (W_{u}[X_{t}\;,{\;h}_{t-1}]+b_{u})$$ (5)

$${\hat{h}}_{t}=tanh(W_{h}\;[X_{t}\;,\;\left(r_{t}⊙h_{t-1}\right)]+b_{h})$$ (6)

$${\;h}_{t}=u_{t}\;⊙{\;h}_{t-1}+(1-u_{t})\;⊙\;{\hat{h}}_{t}$$ (7)

Where ${\;r}_{t}$ and $u_{t}$ are the reset and update gates, ${\;W}_{*}$ and $b_{*}$ are learnable parameters, and ⊙ denotes element-wise multiplication.

Through this mechanism, the model dynamically adjusts graph connectivity according to real-time traffic states, thereby capturing state-aware spatial dependencies that cannot be represented by static adjacency structures. This significantly enhances the robustness of spatial representation learning under highly dynamic traffic environments.

### 3.3. Multi-scale temporal representation learning

Temporal dependencies in traffic systems exhibit both short-term fluctuations and long-range correlations. To effectively capture the complex temporal dependencies inherent in dynamic traffic systems, a Temporal-level Learner is designed to jointly extract local temporal fluctuations and global long-term dependencies from multi-period traffic sequences. Specifically, we integrate a Temporal Convolutional Network (TCN) into the Transformer architecture, enabling a unified hierarchical framework that models both short-range and long-range temporal features while dynamically adapting through meta-learning. This integration empowers the model to learn transferable temporal representations and adapt rapidly to unseen time-domain variations.

Given the traffic sequence $X\in {\mathbb{R}}^{N\times T\times C}$, the TCN first performs 1D dilated convolutions along the temporal dimension to extra_K, the operation at time step t for channel c is formulated as [47–48]:


$$\overset{TCN}{\underset{t,c}{F}}=\sum_{k=1}^{K-1}w_{c,k}\cdot X_{t-d\cdot k,c}+b_{c}$$ (8)

where *d* is the dilation factor, K the kernel size, and $w_{c,k}$, $b_{c}$ are learnable parameters.

This operation enlarges the receptive field without increasing parameter complexity, enabling the model to effectively extract short-term temporal locality. After each convolution, nonlinear activation and layer normalization are applied:


$$F^{\prime TCN}=LayerNorm(\sigma (F^{TCN}))$$ (9)

To ensure compatibility with the Transformer encoder, the TCN output is projected into the model dimension $d_{model}$ through a linear transformation:


$$Q_{TCN}=F^{\prime TCN}{\;W}_{p}$$ (10)

While TCN focuses on localized temporal representation, the Transformer captures global sequential dependencies through multi-head attention. To merge local and global information, we introduce a **cross-attention mechanism**, where the Transformer output $Q_{T}$ serves as the Query, and the TCN outputs provide the Key and Value:


$$Attention(Q_{T},\;K_{TCN},{\;V}_{TCN})=softmax(\frac{Q_{T}\overset{⊤}{\underset{TCN}{K}}}{\sqrt{d_{k}}}){\;V}_{TCN}$$ (11)

This allows the model to selectively attend to relevant local-contextual features within the global temporal scope, enhancing its capacity to model both micro-level periodic variations and macro-level traffic evolution.

### 3.4. Self-supervised meta-learning mechanism

To achieve generalization across heterogeneous regions and temporal domains, SSML-Net integrates a Self-Supervised Meta-Learning (SSML) framework that jointly leverages unlabeled data and task-level adaptation. This paradigm is motivated by the dual objective of leveraging unlabeled data to learn transferable, domain-invariant representations through self-supervision, while concurrently employing meta-learning to optimize model parameters for rapid few-shot adaptation. This dual strategy enables the model to learn domain-invariant representations while maintaining fast adaptability to unseen conditions.

#### 3.4.1. Self-supervised objectives.

We design two auxiliary tasks are designed: temporal reconstruction and contrastive representation alignment. Their objectives are formulated as:


$${ℒ}_{rec}=\sum_{t\in \Omega }\overset{\text{2}}{\underset{\text{2}}{\left\|f_{\theta }({\tilde{X}}_{t})-X_{t}\right\|}}$$ (12)

$${ℒ}_{con}=-\sum_{\left(i,j\right)}\log\frac{\exp(sim(z_{i},z_{j})/\tau )}{\sum_{k}\exp(sim(z_{i},z_{k})/\tau )}$$ (13)

$${ℒ}_{ssl}=\lambda_{\text{1}}{ℒ}_{mask}+\lambda_{\text{2}}{ℒ}_{cont}$$ (14)

where Ω denotes the masked index set, $\tilde{X}$ is the partially observed sequence, $h^{\left(t\right)}$ is the embedding at time t, τ is the hyperparameter used to adjust the model’s sensitivity to differences in similarity, and $\lambda_{\text{1}}$, $\lambda_{\text{2}}$ are balancing coefficients.

#### 3.4.2. Meta-learning parameter update.

The core objective of meta-learning is to leverage previously acquired knowledge to achieve rapid adaptation and efficient generalization to new tasks [49,50]. To this end, we systematically introduce meta-learning strategies into both spatial and temporal levels, thereby equipping the model with adaptive capabilities in dynamically changing traffic environments. Specifically:

- Spatial-level meta-learning captures latent commonalities of road networks across different regions and time periods, enhancing cross-scenario transfer.
- Temporal-level meta-learning focuses on the evolutionary characteristics of traffic flow across multiple time scales, enabling the model to flexibly respond to unexpected events and periodic fluctuations.

The central idea of meta-learning is that the model parameters are not directly optimized in a single task, but are instead obtained through a higher-level “learning-to-update” process. Given a set of tasks $T={\{T}_{\text{1}},T_{\text{2}},...,T_{m}\}$, each task corresponds to a specific city or subnetwork. For each task $T_{i}$, we define its support set $\overset{\sup}{\underset{i}{D}}$ and query set $\overset{que}{\underset{i}{D}}$.

The inner-loop update on the support set is expressed as:


$$\overset{\prime }{\underset{i}{\theta }}=\theta -\alpha \nabla_{\theta }({ℒ}_{pred}(\overset{sup}{\underset{i}{D}};\theta )+{ℒ}_{ssl}(\overset{sup}{\underset{i}{D}};\theta ))$$ (15)

The outer-loop update then aggregates the query losses across tasks and performs meta-parameter optimization:


$$\theta \leftarrow \theta -\beta \nabla_{\theta }({ℒ}_{pred}(\overset{que}{\underset{i}{D}};\overset{\prime }{\underset{i}{\theta }})$$ (16)

where θ denotes the meta-learner parameters, α and β denote the inner and outer learning rates, respectively. The meta-parameters $\theta =\theta_{S}+\theta_{T}$ represent spatial and temporal learners, respectively.

The spatio-temporal self-supervised meta-learning mechanism of SSML-Net operates through a bi-level optimization process. The inner-loop optimization rapidly adapts model parameters to new tasks using limited data, while the outer-loop optimization updates the meta-knowledge across tasks by evaluating generalization performance on validation sets. This synergistic design not only enables the model to capture domain-invariant spatio-temporal patterns through self-supervision but also facilitates fast adaptation to unseen urban scenarios via meta-learning. Consequently, the proposed dual-level meta-optimization empowers SSML-Net to achieve rapid cross-domain adaptation, robust generalization, and stable forecasting accuracy in complex, non-stationary environments.

## 4. Experiments and results analysis

### 4.1. Dataset

We evaluate the predictive performance of the proposed SSML-Net on multiple real-world public transportation datasets, covering two typical scenarios: highway and urban road networks. As shown in Table 1, the experimental datasets include three widely used PeMS subsets (PeMS04/07/08), the METR-LA dataset, and the Beijing Traffic Data dataset. Together, these datasets cover transportation networks across different regions, varying network scales, and diverse topological structures.

**Table 1:** Datasets.

Dataset type	Datasets	Sensors	Edge set	Time range	Time interval
Highway traffic flow	PeMS04	307	340	2018-01-01/2018-02-28	5 minutes
	PeMS07	883	1,025	2012-05-01/2012-06-30	5 minutes
	PeMS08	170	295	2016-07-01/2016-08-31	5 minutes
	METR-LA	207	1,515	2012-03-01/2012-06-30	5 minutes
Urban traffic flow	Beijing traffic data	980	2150	2017-04-01/2017-05-31	5 minutes

In the PeMS system, a large number of fixed induction loop sensors are deployed along the main lanes and ramps of highways, continuously recording traffic conditions with a sampling interval of approximately 30 seconds. To maintain consistency with existing research and reduce the impact of high-frequency noise, this study aggregates the raw 30-second data into 5-minute interval average traffic flow sequences, which serve as input for model training and evaluation. Specifically, PeMS04, PeMS07, and PeMS08 correspond to highway traffic flow monitoring data from three distinct regions in California, while the METR-LA dataset originates from the sensor network of highways in the Los Angeles metropolitan area.

To further validate SSML-Net’s generalization capabilities in urban traffic scenarios, we introduce the Beijing Traffic Data dataset, which captures traffic conditions within Beijing’s urban road network. Compared to the predominantly highway-based PeMS and METR-LA datasets, the Beijing dataset exhibits more complex spatio-temporal patterns of urban traffic, providing a more challenging testing environment for evaluating a model’s robustness and transferability within intricate urban road networks.

During data preprocessing, all datasets are divided into training, validation, and test sets in a ratio of 7:1:2 to ensure the comparability and robustness of the experimental results. In addition, to support meta-learning tasks, each training task is further divided into a support set and a query set, where the model first performs fast adaptation on the support set and then is evaluated on the query set.

### 4.2. Experimental details

#### 4.2.1. Baselines.

This study conducts a comprehensive evaluation the performance of SSML-Net against eight representative baseline models in traffic flow prediction. The selected baseline models encompass three methodological paradigms: (1) In the traditional category, ARIMA [7] offers a classical statistical approach for linear time series analysis, while SVR [22] provides a kernel-based nonlinear regression framework. (2)Among deep temporal models, LSTM [30] captures long-range dependencies via gating mechanisms, the Transformer [32] utilizes self-attention for global context modeling, and Informer [44] enhances efficiency for long-sequence forecasting. (3)Representing spatio-temporal approaches, DCRNN [39] integrates diffusion convolution with encoder-decoder architecture, T-GCN [41] couples graph convolution with gated recurrent units, and STGCN [42] combines spatial graph convolution with temporal convolution for integrated modeling. In addition, ST-MetaNet [51] leverages meta-learning to adaptively parameterize spatio-temporal convolutions based on observed traffic conditions, aiming to capture dynamic and heterogeneous traffic patterns.

#### 4.2.2. Hyperparameter setting.

During the experiments, we train the model using the Adam optimizer with an initial learning rate of 0.001. The maximum number of training epochs is set to 100, and an early stopping strategy is applied: training terminates if the validation loss does not decrease for 10 consecutive epochs. The batch size is fixed at 64, and the input traffic data are normalized using the Min-Max method. The hyperparameter configuration is determined through multiple preliminary experiments, where different parameter combinations are validated and the final choice is made based on model performance on the validation set. For the input–output settings, the model takes 12 consecutive time steps of historical traffic flow data as input and predicts traffic conditions for the next 3, 6, and 12 time steps (corresponding to 15, 30, and 60 minutes, respectively).

All experiments are implemented in PyTorch and run on an NVIDIA GPU with 16 GB of memory. The hardware and software specifications of the experimental environment are provided in Table 2.

**Table 2:** Experimental environment.

Software and hardware configuration	Configuration parameter
CPU	13th Gen Intel(R) Core(TM) i5-13500HX (2.50 GHz)
GPU	INVIDIA GeForce RTX 4050
Programming language	Python 3.12
Deep learning framework	PyTorch 2.5.1

#### 4.2.3. Evaluation metrics.

To quantitatively evaluate the predictive performance of the proposed SSML-Net model and baseline models, this study employs three commonly used metrics in the field of traffic flow forecasting: Mean Absolute Error (MAE), Root Mean Squared Error (RMSE), and Mean Absolute Percentage Error (MAPE). MAE measures the average magnitude of prediction errors and provides a direct indication of the overall prediction accuracy of the model. RMSE squares the errors, making it more sensitive to larger deviations, and is commonly used to assess the robustness of model predictions. MAPE expresses the errors as relative percentages, making it suitable for comparing performance across datasets of different scales. These metrics capture prediction accuracy from different perspectives and provide a comprehensive evaluation of model performance. The definitions of these metrics are as follows:


$$MAE=\frac{\text{1}}{N}\sum_{i=1}^{N}|{\hat{Y}}_{i}-Y_{i}|$$ (17)

$$RMSE=\sqrt{\frac{\text{1}}{N}\sum_{i=1}^{N}({\hat{Y}}_{i}-Y_{i}{)}^{\text{2}}}$$ (18)

$$MAPE=\frac{\text{1}}{N}\sum_{i=1}^{N}|\frac{{\hat{Y}}_{i}-Y_{i}}{Y_{i}}|\times 100\%$$ (19)

Where N denotes the total number of predictions; ${\hat{Y}}_{i}$ and $Y_{i}$ represent the i-th predicted value and the corresponding observed value, respectively.

### 4.3. Traffic flow prediction

We select the classical traffic flow datasets PeMS04, PeMS07, and PeMS08 for experiments, and compare our model with multiple baselines, including traditional statistical methods, deep temporal models, and high-performing spatio-temporal graph-based approaches, for traffic flow prediction at 15-minute, 30-minute, and 60-minute horizons. Repeated experiments show that the proposed SSML-Net outperforms all baseline models in overall performance (see Table 3). As shown in Table 3, the proposed SSML-Net consistently outperforms all baseline models on the three benchmark datasets, PeMS04, PeMS07, and PeMS08, across different prediction horizons of 15 min, 30 min, and 60 min. On the PeMS04 dataset, SSML-Net achieves MAE values of 8.77, 9.30, and 9.41, respectively, which are significantly lower than those of DCRNN (9.48–11.35) and STGCN (9.40–9.97). Furthermore, the MAPE remains below 10.6% for all prediction intervals, demonstrating the model’s superior accuracy and stability. Even as the prediction horizon increases, SSML-Net maintains robust performance, highlighting its strong capability for long-term traffic forecasting.

**Table 3:** Performance comparison on three traffic forecasting datasets.

Metrics		15 min/Horizon 3			30 min/Horizon 6			60 min/Horizon 12		
		MAE	MAPE	RMSE	MAE	MAPE	RMSE	MAE	MAPE	RMSE
PeMS04	ARIMA	14.08	15.36%	17.02	16.57	17.18%	18.89	18.91	21.92%	19.77
	SVR	12.18	16.67%	15.34	14.83	17.26%	16.18	16.09	19.20%	17.92
	LSTM	11.89	14.38%	13.78	13.58	15.63%	14.05	14.05	17.56%	14.78
	Transformer	10.91	13.93%	12.99	12.68	14.49%	13.03	13.09	14.10%	13.47
	Informer	10.50	12.78%	12.35	11.02	13.76%	12.41	11.25	14.96%	13.61
	DCRNN	9.48	11.98%	11.74	10.97	13.60%	12.26	11.35	14.55%	12.64
	T-GCN	9.73	11.47%	11.51	10.61	12.55%	11.94	10.68	12.75%	13.09
	STGCN	9.40	10.88%	11.15	9.09	**11.09%**	12.32	9.97	13.53%	12.60
	ST-MetaNet	8.92	10.80%	11.13	9.43	11.68%	11.60	9.85	12.32%	12.33
	**SSML-Net**	**8.77**	**10.48**%	**10.98**	**9.30**	**11.15**%	**11.24**	**9.41**	**11.97**%	**11.96**
**Metrics**		**15 min/Horizon 3**			**30 min/Horizon 6**			**60 min/Horizon 12**		
		**MAE**	**MAPE**	**RMSE**	**MAE**	**MAPE**	**RMSE**	**MAE**	**MAPE**	**RMSE**
PeMS07	ARIMA	16.87	16.70%	18.95	19.79	19.06%	21.48	22.19	25.19%	25.83
	SVR	15.34	17.61%	16.95	16.93	21.35%	17.81	18.48	24.62%	20.09
	LSTM	13.98	14.30%	14.56	15.17	15.71%	15.63	16.94	17.46%	17.59
	Transformer	13.75	13.98%	15.92	14.56	14.40%	15.29	14.88	15.70%	15.93
	Informer	12.61	13.36%	13.59	12.63	14.36%	14.58	13.66	14.53%	14.83
	DCRNN	11.27	12.38%	13.35	12.92	13.26%	14.16	13.52	14.72%	14.87
	T-GCN	10.95	11.64%	12.33	11.37	12.67%	13.86	12.44	14.78%	14.75
	STGCN	10.47	11.43%	12.36	10.53	12.41%	13.09	11.54	13.75%	13.89
	ST-MetaNet	9.71	10.98%	11.84	10.39	11.22%	12.81	10.96	13.09%	13.62
	**SSML-Net**	**9.28**	**10.69%**	**11.57**	**9.35**	**11.03%**	**12.51**	**10.09**	**12.54%**	**13.03**
**Metrics**		**15 min/Horizon 3**			**30 min/Horizon 6**			**60 min/Horizon 12**		
		**MAE**	**MAPE**	**RMSE**	**MAE**	**MAPE**	**RMSE**	**MAE**	**MAPE**	**RMSE**
PeMS08	ARIMA	14.79	17.21%	17.28	16.89	21.72%	19.61	19.32	20.92%	22.95
	SVR	14.46	18.01%	15.75	15.32	18.70%	17.03	17.53	21.86%	21.91
	LSTM	12.05	15.43%	14.17	12.66	15.59%	16.47	15.18	16.07%	17.26
	Transformer	11.79	14.68%	13.87	12.22	15.42%	14.89	13.60	14.70%	15.35
	Informer	9.38	12.26%	11.65	10.48	13.14%	12.72	11.06	13.01%	12.92
	DCRNN	9.54	9.90%	10.32	9.82	11.56%	11.35	10.32	12.78%	12.95
	T-GCN	9.07	9.17%	10.54	9.67	10.80%	11.06	10.18	10.63%	11.48
	STGCN	8.27	9.34%	11.43	8.70	10.25%	10.85	9.73	10.56%	11.64
	ST-MetaNet	8.39	8.56%	10.14	**8.41**	**9.65%**	10.52	9.42	10.31%	11.94
	**SSML-Net**	**8.08**	**8.29%**	**9.68**	**8.58**	**10.06%**	**10.26**	**9.25**	**10.25%**	**11.53**

On the PeMS07 dataset, the advantages of SSML-Net become even more pronounced. For the 15-minute horizon, SSML-Net achieves MAE = 9.28, MAPE = 10.69%, and RMSE = 11.57, outperforming STGCN (MAE = 10.47, MAPE = 11.43%) and ST-MetaNet (MAE = 9.71, MAPE = 10.98%). At the 60-minute horizon, SSML-Net still achieves MAE = 10.09 and MAPE = 12.54%, indicating remarkable consistency and generalization ability over extended forecasting periods. Similarly, on the PeMS08 dataset, SSML-Net achieves the best overall results, with MAE values of 8.08, 8.58, and 9.25 and MAPE consistently below 10.25%, outperforming Transformer-based models such as Informer (MAE: 9.38–11.06) and graph-based models such as T-GCN (MAE: 9.07–10.18).

Overall, these results demonstrate that SSML-Net effectively captures the nonlinear and dynamic spatio-temporal dependencies inherent in traffic flow data. Its prediction accuracy and stability substantially surpass those of state-of-the-art methods. Compared with representative graph-based models such as DCRNN and STGCN, SSML-Net consistently achieves relatively low MAPE values across different datasets and forecasting horizons, exhibiting performance improvements to varying degrees, confirming the effectiveness of its bi-level decoupled modeling structure, meta-learning–based parameter optimization, and spatio-temporal feature fusion strategy. In conclusion, SSML-Net exhibits superior generalization capability and robustness across diverse datasets and prediction intervals, providing an efficient and scalable solution for high-precision traffic flow forecasting in complex urban environments.

**Fig 4:**
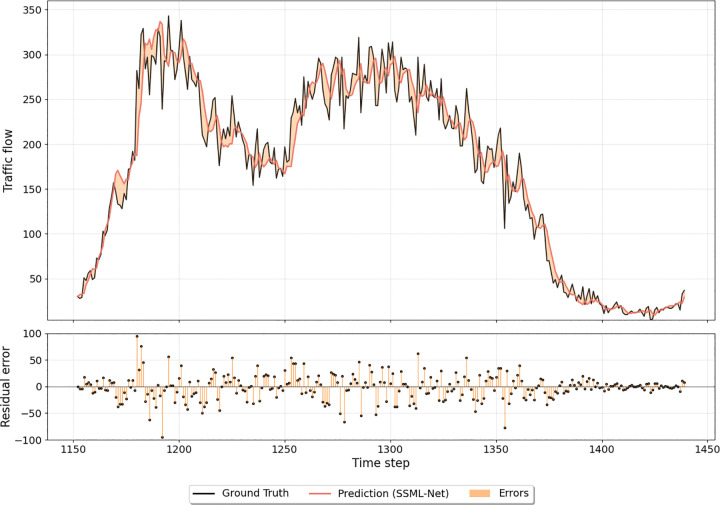
Comparison between SSML-Net traffic flow predictions and observed values over a single weekday.

**Fig 5:**
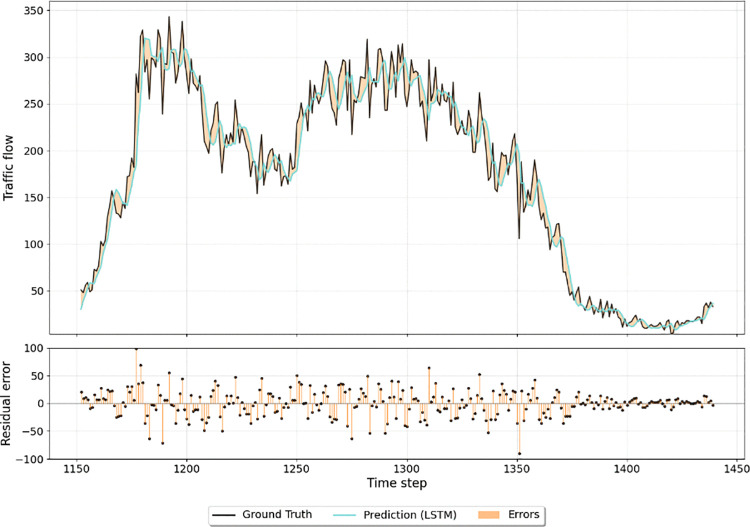
Comparison between LSTM traffic flow predictions and observed values over a single weekday.

**Fig 6:**
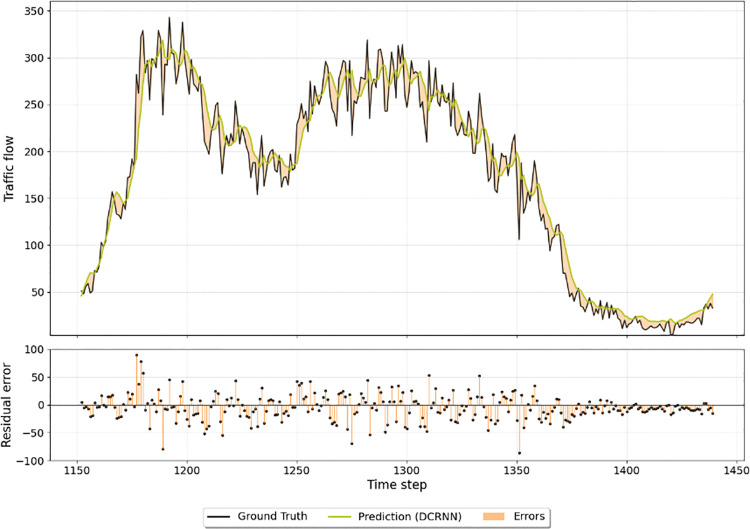
Comparison between DCRNN traffic flow predictions and observed values over a single weekday.

We randomly select the data of one representative node for a single day to conduct a detailed comparative analysis. Fig 4 illustrates the prediction results of SSML-Net against the ground truth, while Figs 5 and 6 display the corresponding outcomes for LSTM and DCRNN, respectively. All three models successfully capture the general temporal trends of traffic flow, demonstrating their capability to learn coarse-grained temporal dependencies. However, when the traffic flow exhibits sharp fluctuations or sudden changes, clear distinctions emerge among the models. SSML-Net produces predictions that respond more promptly and accurately to these variations, maintaining a closer alignment with the ground truth. In contrast, LSTM tends to smooth out rapid transitions due to its limited temporal sensitivity, and DCRNN, while capturing spatial correlations, still exhibits lag and local deviation under abrupt changes.

This superior responsiveness of SSML-Net can be attributed to its decoupled bi-level structure and parallel spatio-temporal learning mechanism, which enable the model to effectively disentangle fast-changing temporal dynamics from stable spatial dependencies. As a result, SSML-Net not only achieves higher fidelity in short-term fluctuation tracking but also maintains stability in long-term trend prediction. These findings further confirm that the proposed architecture enhances the interpretability and robustness of spatio-temporal representation, leading to more accurate and reliable traffic flow forecasting compared with existing baseline methods.

### 4.4. Ablation studies

We conducted a series of ablation studies on the PeMS04 dataset to validate the overall model architecture and to systematically assess the contribution of each functional module. By comparing predictive performance under different component combinations, we further reveal the roles and contributions of each module in the overall modeling process. The specific ablation experiment configurations are as follows:

1) w/o-DynAdj: The static adjacency matrix replaces the proposed state-aware dynamic graph learning to evaluate the impact of adaptive topology refinement on spatial dependency modeling.
2) w/o-TCN: The temporal convolutional network is removed, retaining only the Transformer encoder to examine the effect of local temporal feature extraction.
3) w/o-Trans: The Transformer encoder is omitted while keeping the TCN module to assess the importance of global temporal dependency modeling.
4) w/o-Gate: The meta-learning-driven gated fusion mechanism is replaced with simple concatenation to analyze its role in adaptive spatio-temporal feature integration.

**Fig 7:**
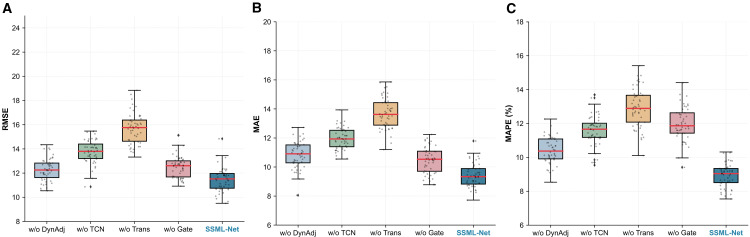
A figure with three subplots: a) RMSE-based predictive performance comparison; b) MAE-based predictive performance comparison; c) MAPE-based predictive performance comparison.

We conduct a series of ablation experiments by comparing four variant models with the proposed SSML-Net, where each variant removes a key architectural component. All models are trained and evaluated under identical experimental settings on the PeMS04 dataset, and the comparative results are illustrated in Fig 7a–7c. The experimental findings demonstrate that the removal of each module leads to a noticeable decline in predictive accuracy, thereby confirming the rationality and necessity of each component in the overall architecture.

Specifically, removing the dynamic adjacency learning (w/o-DynAdj) causes the MAE to increase from 12.34 to 13.89, revealing that the state-aware dynamic graph is essential for capturing the time-varying spatial dependencies and reflecting real-time traffic evolution. When the temporal convolutional network (w/o-TCN) is excluded, short-term prediction accuracy notably declines, suggesting that localized temporal convolutions are essential for capturing rapid fluctuations and fine-grained temporal variations. Conversely, eliminating the Transformer encoder (w/o-Trans) primarily weakens the model’s long-term prediction capability, demonstrating that the global self-attention mechanism effectively captures long-range temporal dependencies and periodic traffic evolution. Finally, removing the meta-learning-based gated fusion mechanism (w/o-Gate) causes a consistent performance drop across all prediction horizons, which indicates that this adaptive fusion strategy plays an indispensable role in integrating spatial and temporal representations in a context-aware manner.

Overall, the ablation studies quantitatively confirm that dynamic adjacency refinement, local–global temporal modeling, and gated fusion collectively contribute to the superior accuracy, robustness, and generalization performance of the proposed SSML-Net architecture.

### 4.5. Few data training

**Fig 8:**
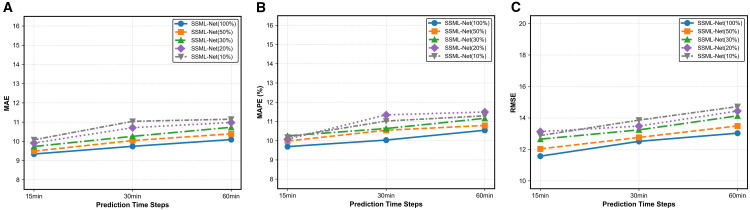
A figure with three subplots: a) Comparison of MAE values across different training data volumes; b) Comparison of MAPE values across different training data volumes; c) Comparison of RMSE values across different training data volumes.

We conduct extensive experiments on the PeMS04 dataset to validate the model’s learning capabilities in low-resource learning environments. Specifically, the model is trained using 100%, 50%, 30%, 20%, and 10% of the original training samples, with the experimental results presented as line charts in Fig 8.

For the 15-minute predictions, the model exhibits strong robustness against reduced training data. MAE increases from 9.34 to 9.49 and 9.73 when using 50% and 30% of the training samples, then increases to 9.91 (20%) and further increases to 10.08 (10%). MAPE increases from 9.69 to 9.98 (50%) and 10.25 (30%), and then exhibits slight non-monotonic fluctuations at lower data ratios (20% and 10%). RMSE generally increases from 11.57 to 13.13 as the training data proportion decreases from 100% to 20%, followed by a slight fluctuation at the 10% data level. These results indicate that short-term predictions exhibit strong robustness against training data reduction, and even with very limited training samples, the errors remain low, demonstrating the model’s robustness in capturing short-term spatiotemporal dependencies.

For the 30-minute predictions, SSML-Net maintains consistent performance under data scarcity. MAE increases from 9.74 to 10.04 and 10.26 at 50% and 30% of training samples, rises further to 10.71 (20%), and then slightly increases to 11.04 (10%). MAPE increases from 10.03 to 11.34 as the training data is reduced to 20%, followed by a slight fluctuation at the 10% data level. RMSE increases progressively from 12.51 to 12.76 (50%), 13.24 (30%), and 13.48 (20%), and then increases to 13.86 (10%), indicating that SSML-Net maintains good stability and effectively captures medium-term temporal patterns even under data scarcity.

For the 60-minute predictions, the model shows controlled performance variation across data regimes. MAE increases from 10.09 to 11.15 as the proportion of training data decreases from 100% to 10%. MAPE gradually rises from 10.54 to 10.79 (50%) and 11.16 (30%), peaks at 11.49 (20%), then slightly declines to 11.29 (10%). RMSE consistently increases from 13.03 to 13.49 (50%), 14.13 (30%), and 14.44 (20%), before falling to 14.72 (10%). Although long-term forecasting exhibits mild fluctuations, error increments remain bounded, and prediction accuracy is retained across all training subsets.

Overall, SSML-Net demonstrates minimal performance degradation across full and intermediate data settings, and retains stable forecasting capability under extreme data scarcity. This underscores the model’s robustness and strong generalization in learning spatiotemporal dependencies across multiple forecasting horizons.

The non-monotonic behavior observed across training subsets can be attributed to several data-specific factors:

(1) Temporal coverage variability: The temporal distribution of different training subsets varies, with some subsets lacking peak hours, holidays, or unusual traffic periods, which affects the performance metrics.
(2) Irregular distribution of extreme events: Outliers or rare traffic conditions are unevenly sampled across subsets, leading to localized error peaks.
(3) Subset quality heterogeneity: Variations in data completeness and noise levels across sampled subsets may non-uniformly affect model performance, though overall error variation remains within an acceptable range.

### 4.6. Investigation of hyperparameter sensitivity

To systematically evaluate the sensitivity of the proposed model to key hyperparameters, we designed multiple sets of controlled experiments examining the impact of attention head count (*K*), SSML-Net layer count (*L*), and batch size on model performance. Experimental results demonstrate significant sensitivity of model performance to these hyperparameters, with distinct optimal configuration ranges identified. The experimental process is as follows:

(1) We systematically evaluated the impact of attention head count on traffic forecasting performance by testing network architectures with varying numbers of attention heads on the PeMS dataset and recording the changes in key evaluation metrics.The influence of attention head count (*K*) on traffic flow prediction effectiveness is presented in Table 4.

**Table 4:** Performance comparison of models in different attention headcounts.

Attention headcounts	MAE	MAPE	RMSE
*K* = 1	9.92	11.20%	12.66
*K* = 2	9.55	11.06%	11.74
*K* = 3	9.43	10.81%	11.26
***K* = 4**	**9.35**	**10.48%**	**10.93**
*K* = 5	9.74	10.92%	11.52

Experimental results indicate that model performance does not exhibit a monotonically increasing trend with the number of attention heads. When *K* increases from 1 to 4, all evaluation metrics show continuous improvement, suggesting that increasing the number of attention heads aids the model in capturing feature information from different subspaces within the data, with optimal performance achieved at *K* = 4. However, when *K* is further increased to 5, all metrics exhibit a slight decline. This phenomenon aligns with the over-parameterisation issue in machine learning, wherein excessive attention heads may induce model overfitting or introduce redundant computational units, thereby diminishing its generalisation capability. Based on the aforementioned analysis, this study ultimately determines *K* = 4 as the optimal configuration for the number of attention heads.

(2) While maintaining fixed parameters such as the number of attention heads (*K* = 3), our system systematically adjusted the number of layers L in the SSML-Net within the spatial correlation module to investigate the impact of network depth on model performance. Comprehensive testing was conducted on the PeMS dataset, with corresponding experimental results summarised in Table 5.

**Table 5:** Impact of the number of SSML-Net layer on model performance.

Layers	MAE	MAPE	RMSE
*L* = 1	10.24	12.75%	13.45
*L* = 2	9.88	11.46%	11.72
***L* = 3**	**9.35**	**10.48%**	**10.93**
*L* = 4	9.61	10.86%	11.18

As observed in Table 5, increasing the number of network layers from *L* = 1 to *L* = 3 yields a marked improvement in model performance, with all metrics showing consistent enhancement. This demonstrates that deepening the network effectively strengthens the model’s representational capacity, enabling it to learn more complex, deep-level features. However, when the number of layers further increased to *L* = 4, all performance metrics deteriorated. Such degradation typically stems from vanishing gradients, exploding gradients, or overfitting issues. That is, excessively deep networks may hinder gradient backpropagation or cause the model to overlearn noise in the training data, thereby weakening its predictive robustness on new data. Consequently, *L* = 3 was confirmed as the optimal configuration for this model’s layer count.

**Fig 9:**
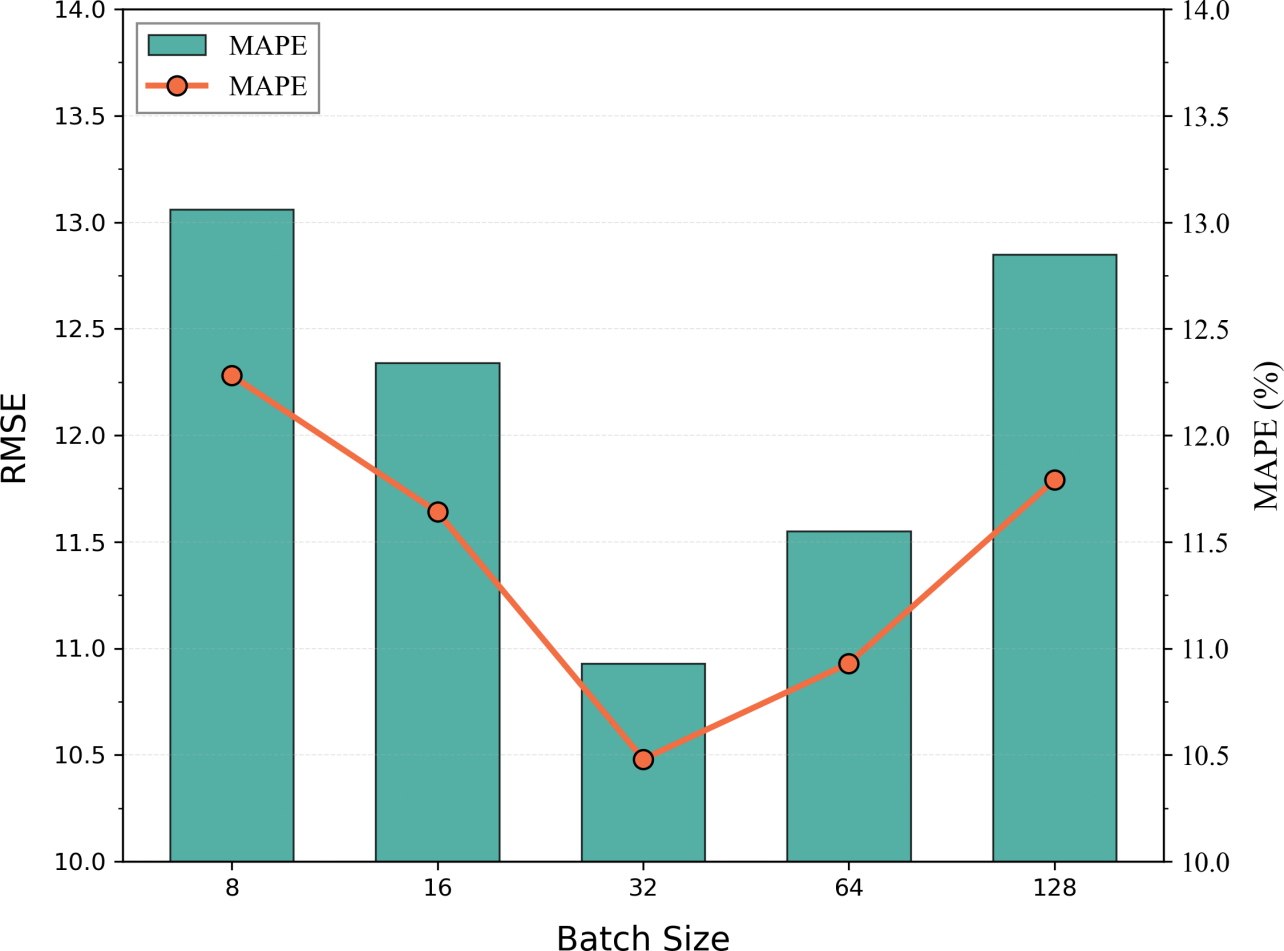
Impact of batch size on model performance.

(3) In parallel, we conducted sensitivity analysis experiments on the critical training hyperparameter of batch size. As shown in Fig 9, the batch size sensitivity experiments reveal that model performance initially increases then declines as batch size grows. When batch size is set to a medium scale, all evaluation metrics achieve optimal values, indicating an effective balance between training efficiency and generalisation capability at this point. Excessively small batch sizes lead to unstable training and significant performance fluctuations; conversely, excessively large batches may cause the model to become trapped in local optima, resulting in diminished generalisation capabilities. Experimental findings demonstrate that a moderate batch size effectively balances the variance and bias in gradient estimation, thereby enhancing overall model performance. Consequently, this study ultimately selected a batch size of 32 as the model’s training configuration.

### 4.7. Training efficiency and generalization

**Fig 10:**
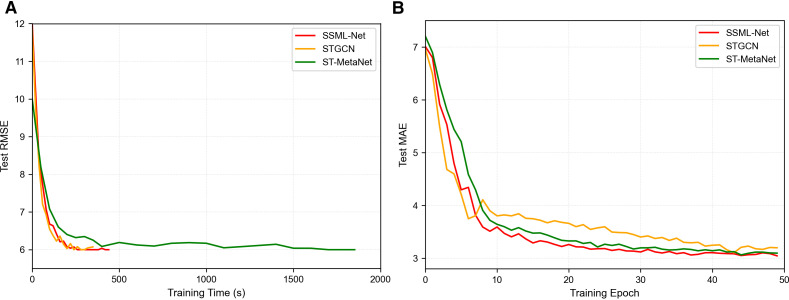
a) Test RMSE versus the training time; b) Test MAE versus the number of training epochs. (Beijing Traffic Data).

To systematically evaluate the performance of the proposed SSML-Net in terms of training efficiency and generalization capability, we conducted comparative experiments against typical spatio-temporal prediction models STGCN and ST-MetaNet on the METR-LA and Beijing Traffic Data datasets. The relevant results are shown in Table 6 and Fig 10.

**Table 6:** Time consumptions of training on the dataset METR-LA/ Beijing Traffic Data.

Dataset (#Number of nodes)	Time Consumption(s)		
	SSML-Net	STGCN	ST-MetaNet
METR-LA (207)	**240.65**	269.67	576.84
Beijing Traffic Data (980)	439.58	**348.98**	1851.37

On the medium-sized METR-LA dataset, SSML-Net completed training in just 240.65 seconds, significantly faster than ST-MetaNet (576.84 seconds) and marginally outperforming STGCN (269.67 seconds). Simultaneously, SSML-Net consistently maintained lower test RMSE/MAE throughout the entire training process (as shown in Fig 10), demonstrating higher training efficiency and a more stable optimization process.

When scaled to 980 nodes (Beijing Traffic Data), SSML-Net’s training time increased only to 439.58 seconds, whereas ST-MetaNet surged to 1851.37 seconds, revealing a significant scalability bottleneck. This disparity primarily stems from SSML-Net’s adoption of an ultra-lightweight dynamic-aware graph module and a single-weight graph convolution structure. This approach avoids the frequent generation and updating of large-scale, high-dimensional meta-parameters required by ST-MetaNet, resulting in smoother computational complexity scaling with node count. Consequently, SSML-Net maintains a significant efficiency advantage even in large-scale road network scenarios.

Regarding generalization performance, Fig 10 demonstrates that SSML-Net achieves faster convergence and lower steady-state error on both datasets. This advantage stems primarily from the organic integration of TCNs with Transformer encoders, which effectively mitigates gradient instability issues inherent in deep recurrent structures. Consequently, the model maintains stable and efficient convergence behavior even when tackling large-scale spatio-temporal sequence modeling tasks.

Furthermore, unlike ST-MetaNet and ST-GDN which primarily rely on supervised loss or graph structure modeling, SSML-Net introduces a self-supervised learning mechanism within a unified spatio-temporal architecture. This explicitly constrains spatio-temporal representations, effectively enhancing the model’s robustness against cross-city distribution shifts and noisy perturbations. Furthermore, the meta-learning strategy adopted by SSML-Net is more memory-efficient, as its task adaptation process occurs solely within a low-dimensional task embedding space rather than requiring extensive parameter reconfiguration of the high-dimensional prediction network. This approach differs from both the reparameterization paradigm of ST-MetaNet and the design philosophy of ST-GCN, which primarily focuses on graph generation mechanisms.

In summary, under identical hardware and training budgets, SSML-Net significantly reduces overall training time while maintaining or even improving prediction accuracy for traffic forecasting tasks across different regions. This demonstrates excellent trainability and stability, better suited for practical city-level deployment.

## 5. Conclusions

In this study, we propose SSML-Net, which accurately captures dynamic spatio-temporal dependencies in traffic flow through dual-layer decoupled modeling and meta-learning-driven adaptive feature fusion. By incorporating self-supervised meta-learning into both spatial and temporal dimensions and introducing meta-learning-driven feature fusion and gated coupling mechanisms, SSML-Net achieves efficient spatio-temporal interaction, rapid adaptation, and strong generalization, demonstrating superior prediction accuracy and robustness in complex traffic scenarios.

We conduct four sets of experiments to comprehensively evaluate the model. (1) Based on the PeMS datasets, we compare SSML-Net with traditional statistical approaches, deep temporal models, and spatio-temporal graph-based meth-ods. The results show that SSML-Net significantly outperforms existing state-of-the-art methods in both predictive accu-racy and stability, demonstrating its ability to effectively capture the inherent nonlinear and dynamic spatio-temporal de-pendencies in traffic flow data. (2) Ablation experiments further validated the effectiveness and necessity of the four core components of SSML-Net. (3) We conduct small-sample ex-periments by training the model with 50%, 30%, 20%, and 10% of the original training data. The results indicate that SSML-Net maintains limited performance fluctuations between the full and reduced datasets, and remains stable even un-der extreme data scarcity. This demonstrates its robustness and ex-cellent generalization ability in modeling short-term, medium-term, and long-term spatio-temporal dependencies. (4) Training efficiency and generalisation performance evaluations conducted using the METR-LA and Beijing Traffic Data datasets demonstrate that SSML-Net exhibits exceptional transfer and generalisation capabilities in cross-domain traffic forecasting scenarios. It not only achieves superior prediction accuracy but also significantly reduces model training costs. By modelling the dynamic spatial dependencies of perceived traffic states, this model surpasses STGCN in performance while avoiding the meta-learning overhead associated with ST-MetaNet.

Despite these promising results, several limitations of this study should be acknowledged. First, the integration of dynamic graph learning, Transformer encoders, and meta-learning mechanisms inevitably increases computational complexity, which may pose challenges for large-scale deployment and real-time applications. Second, although SSML-Net demonstrates strong generalization under data-scarce conditions, its cross-domain transferability across different cities or traffic systems remains an open question.

In future work, we will extend SSML-Net to incorporate multimodal data sources such as weather conditions, traffic incidents, and trajectory information to further enhance its modeling capability. We will also explore domain-specific self-supervised pre-training tasks tailored to traffic data, including traffic event detection, route flow prediction, and incident-aware temporal modeling, aiming to capture traffic dynamics more effectively and provide richer spatio-temporal representations. In addition, model compression and efficiency optimization strategies will be investigated to improve inference speed and practical deployment capability. Moreover, we plan to explore the applicability of the proposed framework to broader spatio-temporal forecasting tasks and cross-domain scenarios, with the goal of developing more transferable and scalable intelligent traffic prediction models.
